# Genotypic and phenotypic features of all Spanish patients with McArdle disease: a 2016 update

**DOI:** 10.1186/s12864-017-4188-2

**Published:** 2017-11-14

**Authors:** Alfredo Santalla, Gisela Nogales-Gadea, Alberto Blázquez Encinar, Irene Vieitez, Adrian González-Quintana, Pablo Serrano-Lorenzo, Inés García Consuegra, Sara Asensio, Alfonsina Ballester-Lopez, Guillem Pintos-Morell, Jaume Coll-Cantí, Helios Pareja-Galeano, Jorge Díez-Bermejo, Margarita Pérez, Antoni L. Andreu, Tomàs Pinós, Joaquín Arenas, Miguel A. Martín, Alejandro Lucia

**Affiliations:** 1 Universidad Pablo de Olavide, Sevilla, Spain; 2 Instituto de Investigación Hospital 12 de Octubre (i+12), Madrid, Spain; 3 Grup de Recerca en Malalties Neuromusculars i Neuropediatriques, Department of Neurosciences, Institut d’Investigació en Ciències de la Salut Germans Trias i Pujol, Universitat Autònoma de Barcelona, Camí de les Escoles, s/n 08916 (Barcelona), Badalona Spain; 4 Servicio de Pediatría, Hospital Universitari Germans Trias i Pujol, Badalona, Spain; 5 Centre for Biomedical Network Research on Rare Diseases (CIBERER), Instituto de Salud Carlos III, Madrid, Spain; 6 Laboratorio de Enfermedades Mitocondriales y Neuromusculares, Hospital 12 de Octubre, Madrid, Spain; 7 Rare Diseases and Pediatric Medicine Group, Galicia Sur Health Research Institute, Complexo Hospitalario Universitario de Vigo (CHUVI), SERGAS, Vigo, Spain; 8 Servicio de Neurología, Hospital Universitari Germans Trias i Pujol, Badalona, Spain; 9 Universidad Europea de Madrid, Madrid, Spain; 10 Departament de Patologia Mitocondrial i Neuromuscular, Hospital Universitari Vall d’Hebron, Institut de Recerca (VHIR), Universitat Autónoma de Barcelona, Barcelona, Spain

**Keywords:** McArdle disease, Spanish patients, Genotype, Phenotype, Glycogenosis type V

## Abstract

**Background:**

We recently described the genotype/phenotype features of all Spanish patients diagnosed with McArdle disease as of January 2011 (*n* = 239, prevalence of ~1/167,000) (*J Neurol Neurosurg Psychiatry* 2012;83:322–8). Several caveats were however identified suggesting that the prevalence of the disease is actually higher.

**Methods:**

We have now updated main genotype/phenotype data, as well as potential associations within/between them, of all Spanish individuals currently diagnosed with McArdle disease (December 2016).

**Results:**

Ninety-four new patients (all Caucasian) have been diagnosed, yielding a prevalence of ~1/139,543 individuals. Around 55% of the mutated alleles have the commonest *PYGM* pathogenic mutation p.R50X, whereas p.W798R and p.G205S account for 10 and 9% of the allelic variants, respectively. Seven new mutations were identified: p.H35R, p.R70C, p.R94Q, p.L132WfsX163, p.Q176P, p.R576Q, and c.244-3_244-2CA. Almost all patients show exercise intolerance, the second wind phenomenon and high serum creatine kinase activity. There is, however, heterogeneity in clinical severity, with 8% of patients being asymptomatic during normal daily life, and 21% showing limitations during daily activities and fixed muscle weakness. A major remaining challenge is one of diagnosis, which is often delayed until the third decade of life in 72% of new patients despite the vast majority (86%) reporting symptoms before 20 years. An important development is the growing proportion of those reporting a 4-year improvement in disease severity (now 34%) and following an active lifestyle (50%). Physically active patients are more likely to report an improvement after a 4-year period in the clinical course of the disease than their inactive peers (odds ratio: 13.98; 95% confidence interval: 5.6, 34.9; *p* < 0.001). Peak oxygen uptake is also higher in the former (20.7 ± 6.0 vs. 16.8 ± 5.3 mL/kg/min, *p* = 0.0013). Finally, there is no association between *PYGM* genotype and phenotype manifestation of the disease.

**Conclusions:**

The reported prevalence of McArdle disease grows exponentially despite frequent, long delays in genetic diagnosis, suggesting that many patients remain undiagnosed. Until a genetic cure is available (which is not predicted in the near future), current epidemiologic data support that adoption of an active lifestyle is the best medicine for these patients.

**Electronic supplementary material:**

The online version of this article (10.1186/s12864-017-4188-2) contains supplementary material, which is available to authorized users.

## Background

Glycogenosis type V [glycogen storage disease type V (GSD V), McArdle disease or *myophosphorylase* deficiency; OMIM® 232,600] is an autosomal recessive disease of carbohydrate metabolism. It is caused by inherited pathogenic mutations in the gene Phosphorylase, Glycogen, Muscle (*PYGM*), encoding the muscle-specific isoform of glycogen phosphorylase, *myophosphorylase*, which catalyzes the breakdown of glycogen into glucose-1-phosphate in this tissue [1]. This myopathy is arguably the prototype of exercise intolerance, which typically consists of acute crises of early fatigue and contractures, occasionally accompanied by rhabdomyolysis and myoglobinuria [1]. The current best diagnostic tool for McArdle disease is genetic testing to determine whether patients are homozygous or alternatively compound heterozygous for pathogenic *PYGM* mutations [1]. Yet the so-called ‘second wind’ phenomenon, that is, marked improvement in tolerance to dynamic exercise (*eg*, bicycling at a constant, submaximal wattage) after 6–10 min of exertion, with subsequent disappearance of previous tachycardia, is a unique characteristic of patients with McArdle disease that is easily measurable [2]. Thus, laboratory assessment of this second wind can be used to support (or discard) the presence of McArdle disease before eventual genetic diagnostic confirmation.

Epidemiologic data available on McArdle disease are relatively scarce and usually limited in sample size [3–7]. We recently described the main genotype and phenotype features of all Spanish patients diagnosed with McArdle disease, as of January 2011 [8]. According to our prior study, reporting on the largest series of McArdle patients published to date (*n* = 239), the prevalence of the disease was ∼1/167,000 Spanish individuals of Caucasian descent. Several caveats were however identified that led us to believe that the actual prevalence of the disease might be higher [8]. A number of patients are likely to remain undiagnosed owing to the rarity of the disease (which is still not well known by many clinicians) or to the mildness of the symptoms in some cases (with no actual interference with daily living activities). Further, many paediatricians are probably unaware of the fact that McArdle disease is to be considered, to a large extent, a paediatric condition, which should expedite diagnosis. In fact, only 4% of Spanish patients were genetically diagnosed during the first decade of life (despite 58% of the total reporting onset of symptoms during childhood), and 47% had not been diagnosed until 30+ years of age [8]. Our previous observations concur with those of recent population estimates by De Castro and colleagues using next-generation sequencing (NGS) of the *PYGM* gene [9]. These authors suggested that the currently accepted prevalence of McArdle disease in Americans of European descendent (~1/100,000) [10] is an underestimate, with the actual disease prevalence being at least 2-fold higher, and thus ≥3-fold higher than the prevalence we recently reported for Spanish patients [8].

The diagnostic protocol followed by the National Health System, where the blood of candidate patients is routinely sent to each of 3 ‘reference’ centres for genetic analysis (*Hospital 12 de Octubre*, Madrid; *Hospital Val d’Hebron*, Barcelona; and *Hospital Meixoeiro*, Vigo), makes it relatively easy to gather data on Spanish McArdle patients. Further, an increasing number of patients with exercise intolerance are referred to our exercise physiology facilities (*Universidad Europea de Madrid* or *Universidad Pablo Olavide*, Seville), which allows us to assess the second wind. Thus, the aim of this study was to update the main genotype and phenotype characteristics, as well as potential associations within/between them, of all Spanish individuals who are currently diagnosed with McArdle disease.

## Methods

We have used a cross-sectional design to perform an update of the main *PYGM* genotype and phenotype data [clinical and laboratory variables (muscle biopsy when available, serum creatine kinase (CK) activity), exercise capacity] of all Spanish individuals diagnosed with McArdle disease, as of December 7th, 2016. The study also has a prospective element, as we have followed-up the 4-year progression of the clinical severity of the disease (see below) in a sub-cohort of 151 patients (*ie*, *n* = 89 from 2006 to 2010, already reported by us [8], and *n* = 62 from 2011 and onwards). The study was approved by the local ethics committees and followed the tenets of the Declaration of Helsinki, 1961.

### *PYGM* genotyping

Mutant *PYGM* alleles were identified in patients’ blood samples using SNaPShot mini-sequencing (ThermoFisher) or polymerase chain reaction and restriction fragment length polymorphism methods [11], followed by: Sanger sequencing of the entire coding region and intron/exon boundaries of the *PYGM* gene [12], or the use of a NGS customised gene panel on a PGM-IonTorrent platform (ThermoFisher), consisting of 35 genes, including *PYGM*, associated with metabolic myopathies. In some cases, analysis of muscle or blood mRNA/cDNA was needed to demonstrate the molecular pathogenicity of a presumed mutant allele, particularly when an alteration of the splicing mechanism was suspected [13, 14].

### Phenotype data

We recorded from clinical histories [or from personal interview with (and direct evaluation of) the patients (in those visiting the aforementioned exercise physiology laboratories for functional evaluation)] data on comorbidities, exercise intolerance, self-reported second wind phenomenon, permanent muscle weakness [15, 16], basal serum CK activity after 1+ days with no exercise (last result available), muscle biopsy results (corroborating lack of staining for *myophosphorylase* and no *myophosphorylase* activity in biochemical analyses), and clinical severity class following the classification originally reported by Martinuzzi et al. [5].

Since autumn 2010, we (AS, AL, MP) have interviewed 151 patients on the progression of their disease within the previous 4-year period to ascertain: (i) improvement (change to a lower severity class in the aforementioned Martinuzzi’s scale), (ii) worsening (change to a higher severity class), or (iii) constant (no change). On the same day of the interview, patients were also asked about their physical activity (PA) habits and were classified as physically active if they followed international PA guidelines, that is, doing ≥150 min/week of moderate-vigorous PA (walking/brisk walking, bicycling, swimming) [17, 18].

Patient’s peak oxygen uptake (VO_2_peak) was determined during cycle-ergometry [19] or treadmill testing until exhaustion (in children) as reported elsewhere [19], and the second wind diagnostic test was performed following the methodology reported by Vissing and Haller [2] using consistently the same equipment (metabolic cart and cycle-ergometer) and under the supervision of the same researchers (AS, AL, MP).

### Statistical analysis

Descriptive data are expressed as frequencies (%) and mean ± standard deviation. We compared phenotype data between genders with the χ^2^ test or unpaired Student’s *t* test. To determine whether the clinical condition of patients deteriorates with aging, we compared patients’ age between the different severity groups with 1-factor analysis of variance (and with Tukey’s test for post hoc comparisons), and between patients showing an improvement in disease progression vs. those showing worsening/no change (with unpaired Student’s *t* test).

We also calculated the odds ratio (OR) and 95% confidence interval (CI) to determine the association: between *PYGM* genotype and disease phenotype/progression, on the one hand, and between PA levels and disease progression, on the other. All statistical analyses were performed using the PASW (v.18.0 for WINDOWS, Chicago) and the level of significance was set at 0.05.

## Results

Compared with the first report on all diagnosed Spanish patients as of January 2011 [8], 94 new patients of Spanish nationality (all of Caucasian descent) have been diagnosed with McArdle disease in only 5 years, to sum a total of 333 patients (183 male). Three patients (all males) included in the previous report [8] have died since January 2011, all due to cardiovascular disease, at the age of 56, 67 and 89 years. As such, the prevalence of McArdle disease in the Spanish (Caucasian) population is now ~1/139,543 persons living.

Table 1 shows the *PYGM* mutational spectrum of Spanish patients, which in essence has not changed in the last years. The genetic analysis showed that ~55% of the mutated alleles harbour the commonest Caucasian stop codon mutation p.R50X, 10% the missense p.W798R mutation (a virtually Spanish private mutation) and 9% correspond to the p.G205S (a relatively common Caucasian mutation). The *PYGM* exons containing more mutations are, in decreasing order, exons 1, 18, 17, 15 and 12. No mutations were found in exons 6–8. Yet, 7 novel *PYGM* mutations were identified: 5 missense mutations, p.H35R, p.R70C, p.R94Q, p.Q176P, p.R576Q, 1 frameshift predicting a premature stop codon (p.L132WfsX163, and 1 splice-site microdeletion mutation c.244-3_244-2CA (Additional files 1 and 2). Of note, all the patients carrying a new mutation exhibited the second wind phenomenon, as assessed by us in the laboratory, and consequently the functional diagnosis of McArdle disease was also proven in these cases. We identified the 2 mutant *PYGM* alleles in all but 3 people (99.1% of total). Although only 1 mutant allele has been identified in these 3 individuals, we also consider them to be patients with McArdle disease because they also experienced the second wind phenomenon in laboratory assessment. The main reason for not having yet detected the second pathogenic mutation in these patients is simply lack of time because diagnosis analyses started only 2 months ago (October 2016).

**Table 1:** *PYGM* mutations identified in all Spanish McArdle patients (*N* = 333)

Type of mutation	N	%
p.R50X (c.148C > T) / p.R50X (c.148C > T)	114	34.2%
p.R50X (c.148C > T) / p.W798R (c.2392 T > C)	29	8.7%
p.G205S (c.613G > A) / p.G205S (c.613G > A)	20	6.0%
p.W798R (c.2392 T > C) / p.W798R (c.2392 T > C)	16	4.8%
p.R50X (c.148C > T) / p.G205S (c.613G > A)	14	4.2%
p.R50X (c.148C > T) / p.K754fsX49 (c.2262delA)	8	2.4%
p.C784X (c.2352C > A) / p.R94W (c.280C > T)	5	1.5%
p.R50X (c.148C > T) / p.R94W (c.280C > T)	6	1.8%
p.R50X (c.148C > T) / p.R602W (c.1804C > T)	3	0.9%
p.R50X (c.148C > T) / p.A660D (c.1979C > A)	3	0.9%
p.R50X (c.148C > T) / p.E383K (c.1147G > A)	3	0.9%
p.G205S (c.613G > A) / c.1768 + 1G > A	2	0.6%
p.R50X (c.148C > T) / c.1768 + 1G > A	4	1.2%
p.R50X (c.148C > T) / p.A365V (c.1094C > T)	3	0.9%
p.R50X (c.148C > T) / p.A55GfsX21 (c.163_167delGCTCT)	2	0.6%
p.R50X (c.148C > T) / p.A704V (c.2111C > T)	4	1.2%
p.R50X (c.148C > T) / p.D534fsX5 (c.1601delA)	2	0.6%
p.R50X (c.148C > T) / p.L5VfsX22 (c.13_14delCT)	3	0.9%
p.R50X (c.148C > T) / p.R194W (c.580C > T)	2	0.6%
p.R50X (c.148C > T) / p.R715W (c.2143C > T)	3	0.9%
p.R50X (c.148C > T) / p.T488 N (c.1463C > A) + p.K215 K (c.645G > A)	2	0.6%
p.R576X (c.1726C > T) / p.G136AfsX159 (c.407G > A)	2	0.6%
p.R771PfsX33 (c.2310_2311dupCC) / p.R771PfsX33 (c.2310_2311dupCC)	2	0.6%
p.W388SfsX34 (c.1162_1169delTGGCCGGTinsA)/p.K754fsX49 (c.2262delA)	2	0.6%
p.W798R (c.2392 T > C) / p.K215 K (c.645G > A)	2	0.6%
p.K609 K (c.1827 G > A) / p.K609 K (c.1827 G > A)	1	0.3%
p. Y733X (c.2199C > G) + p.Y733X (c.2199C > G)	1	0.3%
p.A55GfsX21 (c.163_167delGCTCT) / p.A55GfsX21 (c.163_167delGCTCT)	1	0.3%
p.A660D (c.1979C > A) / p.A660D (c.1979C > A)	1	0.3%
p.E125X (c.373G > T) / p.E125X (c.373G > T)	1	0.3%
p.G174D (C.521G > A) / p.K609 K (c.1287G > A)	1	0.3%
p.G205S (c.613G > A) / p.A365V (c.1094C > T)	1	0.3%
p.G205S (c.613G > A) / p.I83F (c.247A > T)	1	0.3%
p.G205S (c.613G > A) / p.Q176_M177insVQ (c.529-8 g > a)	1	0.3%
p.I83F (c.247A > T)/ p.R94W (c.280C > T)	1	0.3%
p.K754NfsX49 (c.2262delA) / c.2380-1G > A	1	0.3%
p.K754NfsX49 (c.2262delA) / p.K754NfsX49 (c.2262delA)	3	0.9%
p.L116P (c.347 T > C) / p.L116P (c.347 T > C)	1	0.3%
p.L587P (c.1760 T > C) / p.A660D (c.1730A > G)	2	0.6%
p.L5VfsX22 (c.13_14delCT) / p.K754fsx49 (c.2262delA)	1	0.3%
p.M442 K (c.1325 T > A) / p.M442 K (c.1325 T > A)	1	0.3%
p.N134KfsX161 (c.402delC) / p.R491AfsX7 (c.1470dupG)	1	0.3%
p.Q577R (c.1730A > G) / p.A660D (c.1730A > G)	1	0.3%
p.R194W (c.580C > T) + p.E797VfsX18 (c.2385_2386delAA)/p.R194W (c.580C > T) + p.E797VfsX18 (c.2385_2386delAA)	1	0.3%
p.R50X (c.148C > T) / ^a^	4	1.2%
p.R50X (c.148C > T) / c.(1969 + 214)_(2177 + 369)de	1	0.3%
p.R50X (c.148C > T) / c.1827 G > A	1	0.3%
p.R50X (c.148C > T) / c.855 + 5G > A	1	0.3%
p.R50X (c.148C > T) / p.E349K (c.1045G > A)	1	0.3%
p.R50X (c.148C > T) / p.G455R (c.1363G > C)	1	0.3%
p.R50X (c.148C > T) / p.G695R (c.2083G > A)	2	0.6%
p.R50X (c.148C > T) / p.K215 K (c.645G > A)	1	0.3%
p.R50X (c.148C > T) / p.L587P (c.1760 T > C)	1	0.3%
p.R50X (c.148C > T) / p.L5VfsX22 (c.13_14delCT) + p.R324G	2	0.6%
p.R50X (c.148C > T) / p.N685Y (c.2053A > T)	1	0.3%
p.R50X (c.148C > T) / p.Q734HfsX7 (c.211_217dupCGCAGCA)	1	0.3%
p.R50X (c.148C > T) / p.Q755X (c.2263C > T)	1	0.3%
p.R50X (c.148C > T) / p.R576X (c.1726C > T)	1	0.3%
p.R50X (c.148C > T) / p.T488 N (c.1463C > A)	1	0.3%
p.R50X (c.148C > T) / p.T692KfsX30 (c.2075_2076delCCinsAAA)	1	0.3%
p.R50X (c.148C > T) / p.W388SfsX34 (c.1162_1169delTGGCCGGTinsA)	1	0.3%
p.R50X (c.148C > T) / R715W (c.2143C > T)	1	0.3%
p.R50X (c.148C > T) / p.V456 M (c.1366G > A)	1	0.3%
p.R50X (c.148C > T) / p.L354P (c.1061 T > C)	1	0.3%
p.W798R (c.2392 T > C) / p.R590H (c.1769G > A)	1	0.3%
p.Y574X (c.1722 T > G) / p.K609 K (c.1827G > A)	1	0.3%
p.L5VfsX22 (c.13_14delCT) / p.L5VfsX22 (c.13_14delCT)	4	1.2%
p.R50X (c.148C > T) / p.E27AfsX50 (c.78_79delTG)	2	0.6%
p.R50X (c.148C > T) / p.L116P (c.347 T > C)	1	0.3%
c.1092-1G > T / c.2444-3_244-2delCA	1	0.3%
p.R50X (c.148C > T) / p.K609 K (c.1287G > A)	1	0.3%
p.R491Afs (c.1470dupG) / ^a^	1	0.3%
p.W388SfsX421 (c.1162_1169delTGGCCGGT)/p.W388SfsX421 (c.1162_1169delTGGCCGGT)	1	0.3%
p.G205S (c.613G > A) / p.R590H (c.1769G > A)	1	0.3%
p.W798R (c.2392 T > C) / ^a^	1	0.3%
p.R50X (c.148C > T) / p.R490W (c.1468C > T)	1	0.3%
p.R50X (c.148C > T) / p.V456 M (c.1366G > A)	1	0.3%
p.W798R (c.2392 T > C) / c.212_218dup (p.Q73HfsX)	1	0.3%
c.2262delA (p.K754Nfs) / c.244-3_244-2delCA	1	0.3%
p.K754fsX49 (c.2262delA) / c.773-2A > T	1	0.3%
p.Q734HfsX7 (c.211_217dupCGCAGCA) / p.Q734HfsX7 (c.211_217dupCGCAGCA)	1	0.3%
p.R50X (c.148C > T) / p.R576Q	1	0.3%
p.R50X (c.148C > T) / p.L132WfsX153 (c.393delG)	1	0.3%
p.R50X (c.148C > T) / p.Q176P (c.527A > C)	1	0.3%
p.R491Afs (c.1470dupG) / p.R491Afs (c.1470dupG)	1	0.3%
p.R94W (c.280C > T) / p.R94W (c.280C > T)	1	0.3%
p.G135R (c.403G > A) / p.R70C	1	0.3%
c.244-3_244-2delCA / c.1093-1G > T	1	0.3%
p.R50X (c.148C > T) / p.H35R (c.104A > G)	1	0.3%
p.W798R (c.2392 T > C) / p.A365V (c.1094C > T)	1	0.3%

^a^Unidentified mutation in one allele

Phenotype data, and how they compare to our previous report [8], are shown in Table 2. They do not essentially differ between sexes (data not shown, *p* > 0.1 for most comparisons). The mean age of the cohort has now slightly increased since the first report (by 3 years), simply reflecting the aging of the previous 239 patients, who still account for the majority of the cohort. The main clinical features of McArdle disease have remained essentially unchanged with regard to our previous report, except for a slight decrease in the proportion of patients with fixed muscle weakness (25% → 21%) [8]. Thus, virtually all patients have exercise intolerance, which has been accompanied by repeated episodes of myoglobinuria (that patients typically refer to as ‘dark urine’) in half of the cohort. In addition, the vast majority of patients are able to report the second wind phenomenon, which they typically refer to as the ability to resume exercise with attenuated fatigue, tachycardia and myalgia after they take a quick rest. Importantly, this phenomenon was easily measurable in all patients (see Additional file 3 for a representative example), except in 1 child (aged 12 years), which also concurs with our previous observation that, as opposed to adults, the second wind may not be easily detectable in some of the youngest patients [20].

**Table 2:** Main phenotype data in all Spanish McArdle patients (n = 333)

	N with data	Result (men + women)	Main change with regards to previous data (n = 239 patients) [1]
Gender		55% male	↔
Age, years (mean ± SD, range)	333	48 ± 19 (12, 99)^a^	↑
BMI, kg/m^2^ (mean ± SD, range)	132	24.7 ± 4.8 (16, 43)	↔
Familial consaguinity (%)	188	17%	↑
Symptomatic father (%)^a^	201	3%	↔
Symptomatic mother (%)^a^	201	6%	↔
Symptoms’ onset (%)	235		
1st decade		66%	↑
2nd decade		20%	↓
3rd decade		5%	↔
≥4th decade		9%	↔
Genetic diagnosis (%)	275		
1st decade		5%	↔
2nd decade		23%	↔
3rd decade		23%	↔
≥4th decade		49%	↔
Exercise intolerance (%)	272	99.6%	↔
Second wind, self-reported (%)	164	91.5%	↑
Second wind, laboratory- determined (%)	119	99.2%	↔
Fixed muscle weakness (%)	240	21%	↓
Recurrent episodes of myoglobinuria (%)	240	51%	↔
Disease severity (%)^b^	240		
Class 0		8%	↔
Class 1		41%	↔
Class 2		30%	↑
Class 3		21%	↓
Disease progression (%)	151		
Improvement		34%	↑↑
Worsening		28%	↔
Constant		35%	↓↓
Acute renal failure (%)	173	6%	↔
Chronic renal failure (%)	171	1%	↔
Comorbidities (%)			
Diabetes^c^	140	9%	↔
CAD	136	9%	↔
Hypertension	135	11%	↔
Cancer	134	1%	↔
Obesity	127	10%	↔
COPD	131	1%	↔
Serum CK (%) >200 U/L, >1000 U/L	179	98%, 68%	↔, ↓
Biopsy diagnosis (%)	205	100%	↔
Physical activity data (% active)	120	50%	↑↑
VO_2_peak, mLO_2_/kg/min (mean ± SD, range)	120	19.9 ± 6.6 (5.9, 41.5)	↔

Data on 3 patients (all males) who died recently (all after the 6th decade of life, due to cardiovascular disease) are included

Symbols: ^a^ ‘symptomatic’ refers to having the main symptomatic features of McArdle disease (i.e., exercise intolerance with or without myoglobinuria or self-reported second wind)

^b^disease severity class following the classification originally reported by Martinuzzi et al. [2]: 0 = asymptomatic or virtually asymptomatic (mild exercise intolerance, but no functional limitation in any daily life activity); 1 = exercise intolerance, contractures, myalgia, and limitation of acute strenuous exercise, and occasionally in daily life activities; no record of myoglobinuria, no muscle wasting or weakness; 2 = same as 1, plus recurrent exertional myoglobinuria, moderate restriction in exercise, and limitation in daily life activities; 3 = same as 2, plus fixed muscle weakness, with or without wasting and severe limitations on exercise and most daily life activities

^c^diabetes diagnosed based on a glucose tolerance test. Abbreviations: BMI, body mass index; CAD, coronary artery disease; COPD, chronic obstructive pulmonary disease; VO_2_peak, peak oxygen uptake

A laboratory feature that characterises the disease and can further assist in its diagnosis is the typically high levels of the muscle damage marker, serum CK activity, which is above reference limits in nearly all patients and above 2000 U/L in ~2 thirds of them, and is also in accordance with our previous report [8]. Other characteristics also remain essentially unchanged, such as frequency distribution among severity classes [at least when grouping the 2 highest severity classes (2 and 3) together], and with the same percentage (8%) of patients who are virtually asymptomatic during normal daily living (*ie*, belonging to class 0, which denotes exercise intolerance only during strenuous activities or sports participation and absence of myoglobinuria episodes). Also, association with comorbidities induced directly or indirectly by the disease appears low, with very few cases of chronic renal failure and a prevalence of major noncommunicable diseases (diabetes, cardiovascular disease, cancer) that does not appear to differ from that expected for the adult Spanish population. Further, 1 patient has reached remarkable longevity (99 years), supporting the overall benign nature of the disease compared with other glycogenoses.

An important problem that persists with regards to the first series [8] is that, despite most patients reporting onset of exercise intolerance symptoms since childhood, typically in physical education classes and in the school playground, genetic diagnosis has been delayed until much later in life. Among the 94 new patients, genetic diagnosis has not been available until the age of 20+ years in 75% of cases, despite the vast majority (90%) reporting symptoms before that age. Yet a major difference compared with our previous report is an increase in the proportion of patients (i) reporting a 4-year improvement in disease severity (21% → 34%) at the expense of those showing no change (51% → 35%); and (ii) adopting an active lifestyle in recent times (32% → 50%). Indeed, those patients who are physically active are 14-fold more likely to report an improvement after a 4-year period in the clinical course of the disease compared with their inactive peers (OR: 13.98; 95%CI: 5.6, 34.9; *p* < 0.001). In addition, a key fitness and health indicator, VO_2_peak, is significantly (*p* = 0.001) higher in physically active patients (20.7 ± 6.0) than in their inactive referents (16.8 ± 5.3 mL/kg/min).

Age has a detrimental effect on several phenotypic features of the disease. The mean age (57 ± 19 years) of those patients in the highest severity class 3 (that includes presence of fixed muscle weakness) is higher than in those in the lower severity classes 1 (46 ± 19 years, *p* = 0.007) and 2 (41 ± 17 years, *p* < 0.001). Finally, *PYGM* genotype is not significantly associated with any of the phenotype data reported in Table 2 after controlling for sex and age (data not shown). Likewise, *PYGM* is not associated with any phenotype data within the subset of patients reporting a 4-year improvement in disease severity, with a similar proportion of *PYGM* alleles harbouring a missense mutation in this subgroup compared with the whole patient cohort (i.e., ~30% of total number of alleles in both cases).

## Discussion

This is the largest series of patients’ data that is available to date on McArdle disease and as such can provide corroborative or novel insights on this disorder. Despite heterogeneity, mainly in terms of *PYGM* genotype but also of disease severity [with almost 1 in 10 patients being fundamentally asymptomatic in daily living (*ie*, belonging to Martinuzzi’s class 0) vs. 2 in 10 being clearly limited in daily life activities and having fixed muscle weakness (class 3)], several features of the disorder, mainly pertaining to phenotype and laboratory data, are common to the vast majority of patients. This should raise the suspicion of the presence of McArdle disease until genetic confirmation is achieved. These common features include intolerance to strenuous exercise coupled with the second wind phenomenon in almost all patients, as well as basal hyper-CK-emia.

The *PYGM* genotype data shown here are in overall agreement with previous reports on smaller Caucasian cohorts [3, 4, 6, 7, 11, 21–23] with p.R50X/p.R50X and p.R50X/p.W798R combinations accounting for 43% of all *PYGM* genotypes and p.R50X being by far the commonest pathogenic genetic variant. In addition, we have identified 7 novel mutations that must be added to the list of pathogenic variants causing McArdle disease [24]. That heterogeneity of *PYGM* genotypes does not account for heterogeneity in the clinical manifestation of the disease is in agreement with previous studies showing no genotype-phenotype correlation [4, 6, 22]. In fact, in our series, biochemical analysis of muscle biopsies consistently showed null *myophosphorylase* activity and most reported *PYGM* mutations have functional consequences, with many actually resulting in no gene transcript levels owing to a protective intracellular mechanism, the so-called nonsense-mediated decay, which degrades transcripts that contain premature termination codons [25]. By contrast, regular PA is likely the main modulator of the phenotype manifestation of the disease, which explains the heterogeneous presentation of the disorder among patients despite all having the same defect at the muscle molecular level, that is, complete inability to metabolize glycogen stores.

A promising novel result is that a growing number of Spanish patients (around one half of them) are now adopting a relatively active lifestyle, which we believe is especially important when considering the strong, positive association between regular PA and a favourable progression of clinical symptoms. These results support and extend the findings from our previous report [8]. This recent tendency reflects, at least in part, the fact that patients are now following our recommendations [notably, during educational talks given by us (AL, AS, GNG, TP) to the patients in each yearly meeting of the Spanish Association of Glycogenosis Patients] to perform low-moderate intensity PA regularly. Furthermore, VO_2_peak was significantly higher in the physically active patients. Indeed, 8 physically active adult patients (vs. only 2 inactive adult patients) have a VO_2peak_ ≥ 8 METs (where 1 MET = resting metabolic rate or 3.5 mL O_2_/kg/min for most humans). This is also an important finding because 8 METs is the minimum threshold for optimal health, above which the risk for cardiovascular mortality in adults is significantly reduced compared with lower values [26]. Our findings on PA also support previous studies showing the benefits of regular, low-moderate intensity exercise training (bicycling, brisk walking) during 8 [19] to 14 weeks to increase the VO_2peak_ of McArdle patients [27].

We believe an important challenge identified by us here as well as previously [8] is the fact that diagnosis of the disease is usually delayed until adulthood despite symptoms frequently occurring since childhood. Misdiagnosis might also be as a potential cause of delayed diagnosis of McArdle disease [28]. This undoubtedly calls for a better characterisation and monitoring of the disease from childhood and adolescence. The possibility of diagnosing McArdle disease should be clearly mentioned in the specific educational training of paediatricians because early diagnosis would favour implementation of regular PA habits (with carbohydrate ingestion and gradual warm-up before strenuous activities) since early childhood (coupled with a diet rich in complex carbohydrates to ensure constant availability of glucose to working muscles) [29]. In this regard, all affected children report problems during physical education classes because the latter usually involve strenuous exercises, such as running, which is particularly painful and fatiguing for all of them compared to their healthy peers. Early diagnosis is particularly important when considering that practice of regular, low-moderate PA usually tracks from childhood to adolescence and from adolescence into adulthood, thereby laying the foundation for a healthy lifestyle over life [30]. As for diagnostic tools, we believe that objective assessment of the second wind followed by genetic analyses using blood samples (quick screening initially for the three most prevalent mutations, p.R50X, p.W798R and p.G205S, which account, alone or in combination, for ~74% of all *PYGM* mutant alleles, and then, whenever needed, searching for further mutations by Sanger sequencing or NGS of the *PYGM* gene) is the most efficient strategy, with no need for performing unpleasant muscle biopsies unless it is a requirement to identify the genetic alteration or to validate the molecular consequences of novel mutations in the *PYGM* gene. Other tests that were traditionally implemented, such as electromyography recordings or the ischaemic forearm test are probably not useful anymore. A question that remains unanswered however is pregnancy outcomes in all women, especially which is the best way of delivery, whether vaginal or caesarean.

## Conclusions

The reported prevalence of McArdle disease seems to grow considerably despite genetic diagnosis being frequently delayed until adulthood and onset of symptoms occurring since childhood. Thus, awareness of this disease and monitorisation is still insufficient, especially among paediatricians. Until a genetic cure is available (which is not predicted in the near future), the current epidemiologic data support that adoption of an active lifestyle is the best medicine for these patients.

## Additional files

Additional file 1: Distribution of pathogenic mutations causing McArdle disease in Spanish patients by exon/intron. (PDF 24 kb)

Additional file 2: Frequency distribution of pathogenic PYGM mutations by exon. (PDF 11 kb)

Additional file 3: Example of second wind assessment in one adult patient. (PDF 253 kb)
