# Supplementary material for: Genotypic and phenotypic features of all Spanish patients with McArdle disease: a 2016 update
Source: BMC Genomics. 2017 Nov 14;18(Suppl 8):819. doi: 10.1186/s12864-017-4188-2 (PMC5688471; doi:10.1186/s12864-017-4188-2)
Supplement: Supplementary file 1 — Distribution of pathogenic mutations causing McArdle disease in Spanish patients by exon/intron. (PDF 24 kb) [file 12864_2017_4188_MOESM1_ESM.pdf]

Additional file 1. Distribution of pathogenic mutations causing McArdle disease in Spanish patients by exon/intron.

**Localization**	**Mutations**
**Exon 1**	p.R50X (c.148C>T), p.L5VfsX22 (c.13_14delCT), p.E27AfsX50 (c.78_79delTG), **p.H35R (c.104A>G)**, p.A55GfsX21 (c.164_168delCTCTG), **p.R70C (c.208C>T)**, p.Q73HfsX7 (c.212_218dupCGCAGCA)
**Exon 2**	p.I83F (c.247A>T), **p.R94Q (c.281G>A)**, p.R94W (c.280C>T)
**Exon 3**	p.L116P (c.347T>C), p.E125X (c.373G>T), **p.L132WfsX163 (c.393delG)**, **p.N134KfsX161 (c.402delC)**, **p.G135R (c.403G>A)**, p.G136AfsX159 (c.407delG)
**Exon 4**	**p.G174D (c.521G>A)**, **p.Q176P (c.527A>C)**, **p.Q176P (c.527A>C)**
**Exon 5**	p.G205S (c.613G>A), p.R194W (c.580C>T), p.K215K (c.645G>A)
**Exon 6**	
**Exon 7**	
**Exon 8**	
**Exon 9**	**p.L354P (c.1061T>C)**, **p.E349K (c.1045G>A)**
**Exon 10**	p.A365V (c.1094C>T), p.E383K (c.1147G>A), p.W388SfsX34 (c.1162_1169delTGGCCGGTinsA)
**Exon 11**	p.M442K (c.1325T>A), **p.G455R (c.1363G>C)**, p.V456M (c.1366G>A)
**Exon 12**	**p.T488N (c.1463C>A)**, **p.R490W (c.1468C>T)**, p.R491AfsX7 (c.1470dupG)
**Exon 13**	p.R534VfsX5 (c.533delA)
**Exon 14**	**p.Y574X (c.1722T>G)**, **p.Q577R (c.1730A>G)**, **p.R576Q (c.1727G>A)**, p.R576X (c.1726C>T), p.L587P (c.1760T>C)
**Exon 15**	p.R590H (c.1769G>A), p.R602W (c.1804C>T), p.K609K (c.1827G>A)
**Exon 16**	**c.(1969+214)_(2177+369)del**
**Exon 17**	p.A660D (c.1979C>A), **p.N685Y (c.2053A>T)**, **p.T692KfsX30 (c.2075_2076delCCinsAAA)**, **p.G695R (c.2083G>A)**, p.A704V (c.2111C>T), p.R715W (c.2143C>T)
**Exon 18**	p. Y733X (c.2199C>G), p.K754NfsX49 (c.2262delA), **p.Q755X (c.2263C>T)**, p.R771PfsX33 (c.2310_2311dupCC)
**Exon 19**	p.C784X (c.2352C>A)
**Exon 20**	p.W798R (c.2392T>C), **p.E797VfsX18 (c.2385_2386delAA)**
**Intron 2**	**c.244-3_244-2CA**
**Intron 4**	**c.528-8G>A (p.Q176_M177insVQ)**
**Intron 6**	**c.772-2A>T**
**Intron 7**	**c.855+5G>A**
**Intron 9**	c.1092-1G>T
**Intron 14**	c.1768+1G>A
**Intron 19**	**c.2379-1G>A**

New, previously unreported mutations are in bold and mutations only found in one patient are in grey color.
