# Supplementary material for: Genotypic and phenotypic features of all Spanish patients with McArdle disease: a 2016 update
Source: BMC Genomics. 2017 Nov 14;18(Suppl 8):819. doi: 10.1186/s12864-017-4188-2 (PMC5688471; doi:10.1186/s12864-017-4188-2)
Supplement: Supplementary file 2 — Frequency distribution of pathogenic PYGM mutations by exon. (PDF 11 kb) [file 12864_2017_4188_MOESM2_ESM.pdf]

						**TOTAL**
**Ex 1** 19.65%	+ **Ex 17** 10.98%	+ **Ex 18** 15.60%			= 46.23%	**46.23%**
**Ex 2** 8.09%	+ **Ex 15** 7.51%				= 15.6%	**61.83%**
**Ex 3** 5.78%	+ **Ex 10** 5.78%	+ **Ex 12** 4.05%	+ **Ex 14** 5.78%		= 21.39%	**83.22%**
**Ex 5** 5.21%	+ **Ex 11** 2.89%	+ **Ex 19** 2.31%			= 10.41%	**93.63%**
**Ex 4** 1.16%	+ **Ex 9** 1.16%	+ **Ex13** 1.16%	+ **Ex16** 0.58%	+ **Ex20** 2.31%	= 6.37%	**100%**

Supplemental file 2. Frequency distribution of pathogenic *PYGM* mutations by exon. Abbreviation: Ex, exon
