# Supplementary material for: Genotypic and phenotypic features of all Spanish patients with McArdle disease: a 2016 update
Source: BMC Genomics. 2017 Nov 14;18(Suppl 8):819. doi: 10.1186/s12864-017-4188-2 (PMC5688471; doi:10.1186/s12864-017-4188-2)
Supplement: Supplementary file 3 — Example of second wind assessment in one adult patient. (PDF 253 kb) [file 12864_2017_4188_MOESM3_ESM.pdf]

Supplemental file 3. Example of second wind assessment in one adult patient.

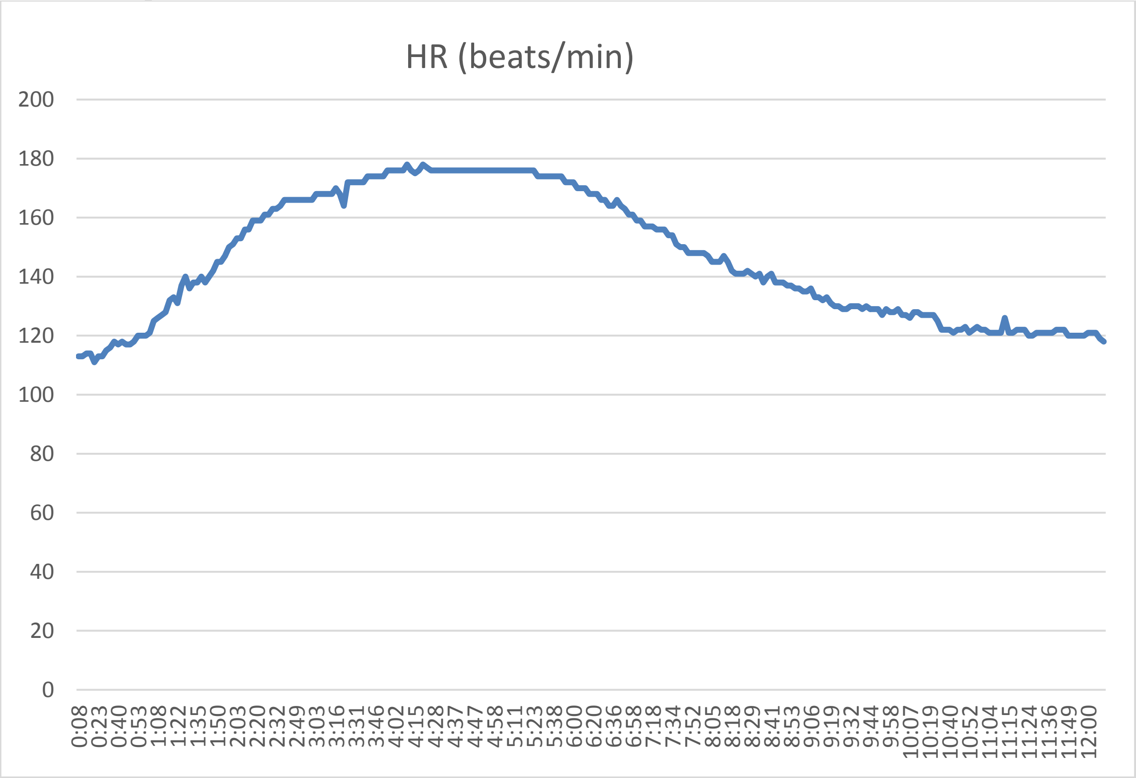

Time (min: seg)

The test consists of a 15-minute bout of cycle-ergometer exercise at a wattage eliciting a heart rate value of 60-70% of the predicted maximum heart rate [i.e., 220 beats/minute *minus* age (in years)] [1]. The patients typically show a decrease in early exertional tachycardia (from ~175 to ~120 beats/min in this example) starting after around 6 minutes of the test. This test only requires a cycle-ergometer and use of a heart rate meter.

1. Vissing J, Haller RG: **A diagnostic cycle test for McArdle's disease**. *Ann Neurol* 2003, **54**(4):539-542.
